# Histological Study of Induced Incisions on Rabbits' Tongues with Three Diode Lasers with Different Wavelengths in Continuous Mode

**DOI:** 10.1155/2018/2691942

**Published:** 2018-05-02

**Authors:** Salwa Yammine, Edgard Jabbour, Sami El Toum, Antoine Cassia

**Affiliations:** ^1^Department of Restorative Dentistry and Endodontic, Faculty of Dentistry, Lebanese University, Beirut, Lebanon; ^2^Department of Oral Medicine and Maxillofacial Radiology, Faculty of Dentistry, Lebanese University, Beirut, Lebanon

## Abstract

**Objective:**

Diode lasers have multiple indications in everyday dental practice. They allow carrying out incisions, coagulation of soft tissue, and Low-Level Laser Therapy. The goal of this study is to compare histologically the tissue interaction zones and edges of an induced laser incision on rabbits' tongues with three different wavelengths of 810, 940, and 980 nm in continuous mode.

**Methods:**

Fourteen male rabbits were divided into six groups. Each animal underwent three incisions of 10 mm length on the right ventral face of the tongue, carried out in continuous mode with three diode lasers with different wavelengths of 810, 940, and 980 nm. Rabbits were sacrificed at 0, 1, 2, 6, and 15 hours and 14 days. Five rabbits were sacrificed at 0 hours and 2 hours and one rabbit was sacrificed at 1, 6, and 15 hours and at 14 days. The appearance of neutrophils marked the onset time of the inflammatory reaction. Histological study of the incisions was chosen to evaluate the edges and to measure the depth and width of carbonization and necrotic and inflammatory zones. Healing was evaluated at 14 days. Friedman test was used to assess statistical differences between groups.

**Results:**

In the experimental adopted conditions, the carbonization zone was marked by degradation of vacuoles and an elongation of nuclei and was observed on the edges of incisions. Carbonization and necrotic and inflammatory zones were measured for rabbits sacrificed at 0, 1, 2, 6, and 15 hours but the onset of inflammation zone marked by the infiltration of neutrophils did not appear before 6 hours. The neutrophils infiltration was higher at 15 hours than at 6 hours. Complete healing was shown at 14 days. According to the time for the regularity of the edges, the interpretation was qualitative without a statistical test. The statistical analysis of the three different diode lasers in this study showed nonsignificant difference between the different groups for the depth (*p* = 0.121) and width (*p* = 0.376) of the incisions, the carbonization zone (*p* = 0.692), and the necrotic zone (*p* = 0.223). For the inflammation zone at 6 and 15 hours, statistical analysis was not carried out; only one rabbit was enough to evaluate onset of neutrophils infiltration and to compare its density for 6 and 15 hours.

**Conclusion:**

These results indicate that diode laser used in the continuous mode is predictable for induced incision. The use of three diode lasers with different wavelengths of 810, 940, and 980 nm did not reveal a significant statistical difference according to depth and width of the incision and for carbonization and necrotic zone. The appearance of neutrophils was marked between 4 and 6 hours and was higher at 15 hours.

## 1. Introduction

Today, the laser has many indications in routine dental practice. In oral medicine, diode laser can be used for biopsy, coagulation, and Low-level Laser Therapy. The purpose of biopsy is to get a specimen with a sharper and less carbonized edge that permits pathological illustration of the limit. Historically, the first laser was developed in 1960 by Maiman [1]. After that, lasers have been used in medicine and surgery.

Lasers are classified according to the active substance that is stimulated. In the diode laser, the active medium is a semiconductor. This innovative technology has enabled the realization of a compact and low-cost laser [2–4]. There are several wavelengths for the laser diode, but the most used in dentistry are Gallium-Aluminium-Arsenide (GaAlAs) (810 nm), Gallium-Arsenide (GaAs) (940 nm), and Indium-Gallium-Arsenide (InGaAs) (980 nm). These wavelengths are well absorbed by hemoglobin and melanin and less absorbed by water [5]. With these lasers, penetration depth of biological tissue is between 0.5 and 3 mm [6–8].

Diode lasers can be used, as clinically indicated, in continuous or pulsed mode and in contact and noncontact mode [9, 10]. The target tissue absorbs laser energy and converses it to thermal effect with temperature increases. Depending on the laser parameters and optical properties of the tissue, the tissue temperature rises differently with multiple zone effects. The tissular reaction zones involved vaporization, carbonization, and necrotic and reversible inflammatory zones. Incisions are accomplished, wherein the temperature is more than 100°C and vaporization of intra- and extracellular water is produced, and tissue ablation is achieved [11–13].

Water absorption is the critical factor influencing the energy conversion of surgical lasers that operate in the infrared range. Most tissues are composed of 60–80% water, so the degree of water absorption determines the ability of the laser to penetrate biological tissue. It is necessary to identify the degree of water absorption for each wavelength [6].

A universal laser does not exist; clinical applications will depend on the chosen wavelength. Lasers currently available in dentistry have different radiation-material interaction [14]. The objective of this study is to compare the edges of an induced incision, on the lingual ventral mucosa of the rabbit, with three wavelength diode lasers of 810, 940, and 980 nm used in dentistry in continuous mode. Histological analysis permits the evaluation of the depth and width of the induced incision and the carbonization and necrotic and inflammation zones of reactional tissue.

## 2. Materials and Methods

Fourteen male rabbits of local strain* Oryctolagus cuniculus domesticus, *aged between three and four months and weighing between 1.5 and 2.5 kg, were used. These animals came from conventional breeding at Laboratory of Physiology, Faculty of Medical Sciences (Lebanese University). They were housed in pairs in wire cages (50/75/100 cm) with removable bottom, equipped with a feeder and a waterer lollipop. The animals were kept at a temperature of 21 ± 2°C and relative humidity of 60 ± 5% with a 12 h/12 h light/dark cycle. They received a standard diet and water ad libitum. These animals were treated according to the standard norms imposed by the European Union (86/609/EEC).

**Figure 1:**
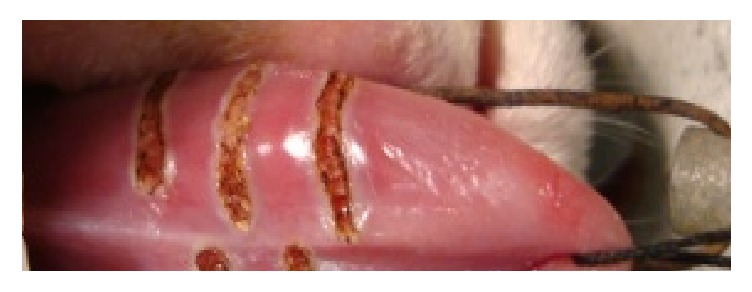
Induced incisions with three different wavelength diode lasers of 810, 940, and 980 nm on the ventral side of the tongue of the rabbit; from top of the tongue, 980, 940, and 810 nm laser-induced incisions.

The fourteen rabbits were divided into six groups. They received an anesthetic dose of 60 mg/kg of Nembutal® (sodium pentobarbital, Abbott Laboratories, Inc., Chicago, USA) by intraperitoneal administration. After sedation, the tongue was pulled out with a suture black silk 4/0 (Ethicon, US) according to the point of the tongue. Three diode lasers of different wavelengths were used; the first one is LaserSmile® with wavelength of 810 nm (group C1), the second one is ezlase® with wavelength of 940 nm (group C2), and the third one is HOYA ConBio® with wavelength of 980 nm (group C3). Each rabbit underwent three incisions on the ventral surface of the tongue, respectively, with the three lasers used by random position. The power used is two watts in continuous mode delivered with an optical fiber of 400 *μ*m. The tip of the fiber was “initiated” by using a blue articulating paper with 1.5 W pulsed 30 ms/30 ms. The fiber was also marked up to 2 mm in length to permit a gauge when performing incision. Each incision had 10 mm length and 2 mm depth and was performed using the activated portion of the fiber as a marker. The position of each relative to the incision or the tip of the tongue has been recorded to recognize the incision in histological sections (Figure 1). A lethal dose of 100 mg/kg was administered at 0 h, 1 h, 2 h, 6 h, 15 h, and 14 days after application of the laser on the tongue and the tongue was immediately dissected and introduced in a solution of 10% formol. The first five rabbits were sacrificed at 0 hours, one rabbit was sacrificed at 1 hour, five rabbits were sacrificed at 2 hours, and one rabbit was sacrificed at 6 and 15 hours and 14 days, respectively.

**Figure 2:**
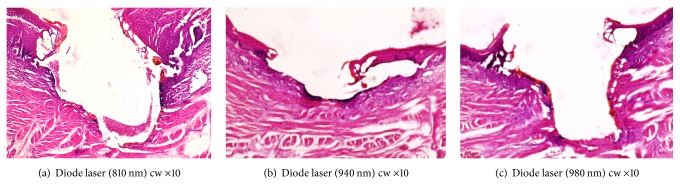
Histological sections (a), (b), and (c) of incisions made with diode lasers (810 nm, 940 nm, and 980 nm) cw: carbonization, the presence of dense eosinophilic layers (denatured collagen) and vascular changes (x10, H&E staining).

Specimens were included in paraffin blocks, and sections of 3 microns in series were realized and stained with hematoxylin-eosin. The histomorphometric measurements were done under a light microscope (Olympus Bx 4500) connected to a digital camera (Nikon Coolpix 4500) at a magnification of x4, x10, and x40 (Figure 2). Three sections per tongue for a total of twelve samples were analyzed manually with software UTHSCSA ImageTool 3.00. The length and width of the incisions and the diameter of each region were measured in each section after inclusion of specimens in paraffin. The specimens were examined separately by two double-blinded histopathologists, and the agreement was reached by estimating the histological changes due to thermal damage by laser.

## 3. Statistical Analysis

The Statistical Package for the Social Sciences (SPSS for Windows, version 20.0, Chicago, IL, USA) was adopted to perform the statistical analysis of data. The variables to be measured were the depth and width of the incisions, the carbonization area, and the necrotic area. Means and standard deviations were reported for each variable.

The edges of the incisions, the inflammatory reaction, and healing were controlled qualitatively by histological examination without statistical analysis.

Friedman test was used for statistical comparison between the three groups of laser transmission: C1 (continuous mode wavelength of 810 nm), C2 (continuous mode wavelength of 940 nm), and C3 (continuous mode wavelength of 980 nm). A *p* value less than 0.05 was deemed statistically significant. Rabbits sacrificed at 0, 1, and 2 hours were pooled for the statistical evaluation of the depth and width of the incisions, carbonization, and necrotic zones. Graphs (error bars representing each variable's mean and its 95% confidence interval) were drawn to show differences between groups.

## 4. Results

The sections from the ventral face of the rabbit's tongue using the diode laser (810, 940, and 980 nm) showed a similar thermal denaturation, such as carbonization, characterized histologically by degradation vacuoles, pyknosis, karyolysis, karyorrhexis, and elongation of nuclei at the edges of the incisions (Figure 2).

**Figure 3:**
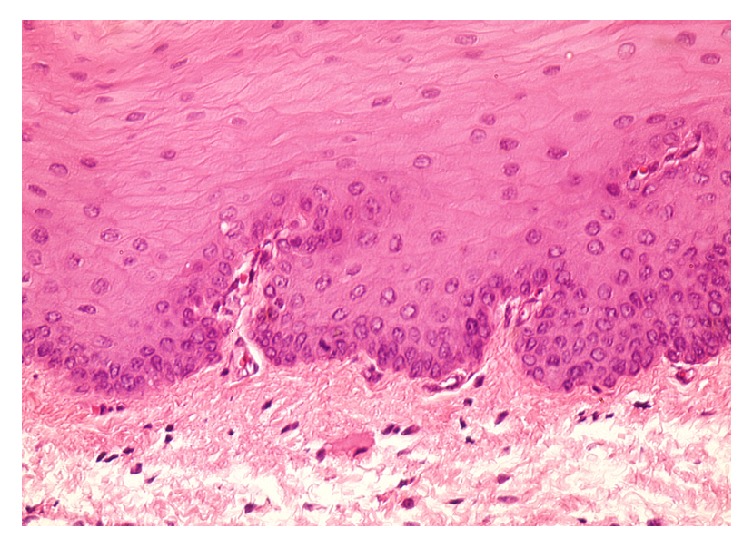
Histological view showed the presence of congestion (neutrophils and monocytes), edema (spaced fibers and cells), and leukocyte diapedesis of PMN after 6 h (x40, H&E staining).

The incisions made in continuous mode showed more significant inflammatation at 15 hours than at 6 hours, which was marked by a high concentration of neutrophils, and healing was completed at 14 days. Groups C1, C2, and C3, sacrificed, respectively, at 0 hours, 1 hour, and 2 hours, noted the absence of polymorphonuclear (PMN) unlike those sacrificed at 6 and 15 hours (Figure 3).

The carbonized region (necrosis) is characterized by the absence of the cores, whereas the coagulated region is characterized by elongated cells with pycnotic nuclei and homogenized cytoplasm. A swelling layer with PMN infiltration was noted in the underlying conjunctiva (Figure 2).

Rabbits well tolerated the incision with the laser without intraoperative or postoperative adverse effects. The induced incisions had complete healing after 14 days without functional loss.  Table 1 illustrates the result of the measures of different parameters.

**Figure 4:**
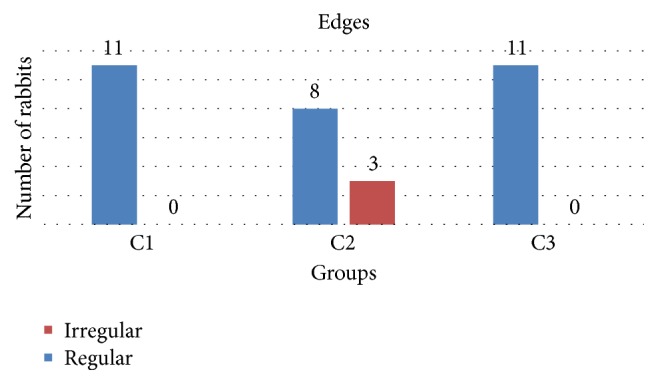
The regular edges were sharper and more defined and straight in C1 and C3 groups in continuous mode unlike the jagged edges in continuous mode in C2 group.

The* edges of the incisions* were microscopically evaluated and classified into two groups: an incision with regular edges where well-marked margins edges were noted, and irregular edges were poorly defined edges, interspersed on margins. The interpretation of the values was qualitative for this parameter. With diode laser with wavelength of 940 nm, three of the 11 rabbits controlled showed an irregular edge (Figure 4).

**Figure 5:**
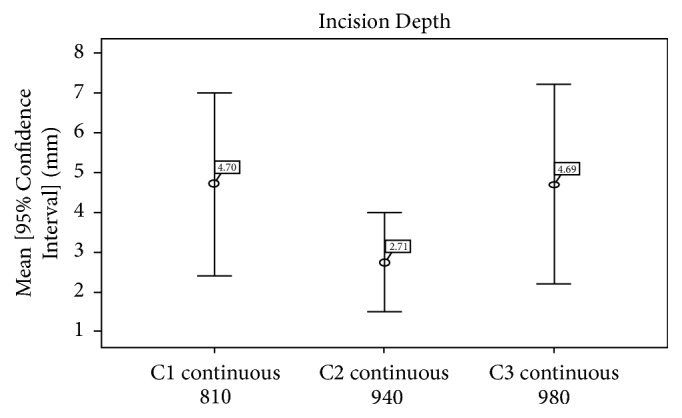
Average incision depths of groups C1, C2, and C3.

The* depth of the incisions* induced by diode laser with wavelengths of 810 nm, 940 nm, and 980 nm showed that the average of the depth was smaller with group C2 (940 nm) (Figure 5). Statistically, there was no significant difference between the three groups, C1, C2, and C3 (*p* = 0.121) (Table 2).

**Figure 6:**
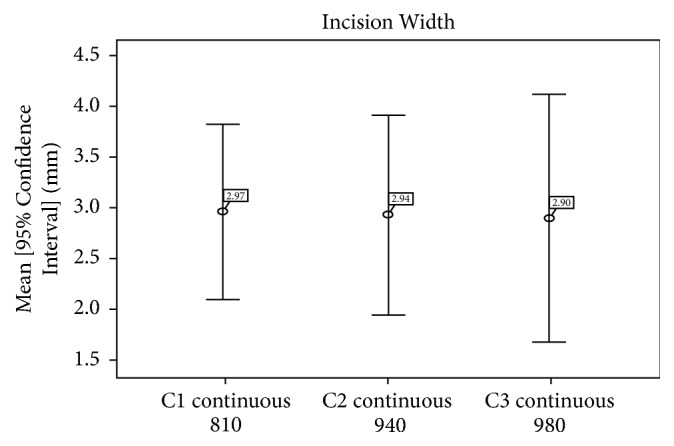
Average incision widths of groups C1, C2, and C3.

The* width of incisions* was smaller in group C3 (980 nm) in comparison with groups C1 and C2 (Figure 6), but the difference was not statistically significant (*p* = 0.376) (Table 2).

**Figure 7:**
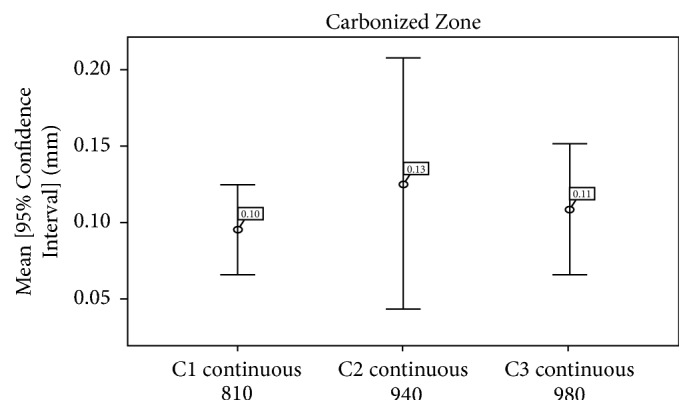
Average carbonized zones of groups C1, C2, and C3.

*The carbonization zone* in this study was more marked with diode laser with wavelength of 940 nm (Figure 7) but did not reveal any statistically significant difference between the three groups (*p* = 0.692) (Table 2).

**Figure 8:**
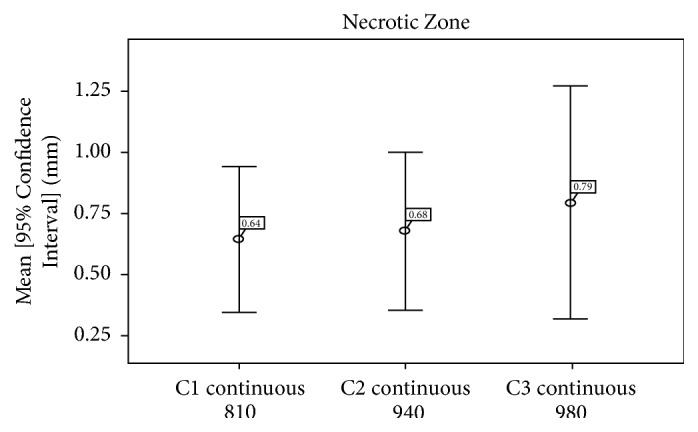
Average necrosis zones of groups C1, C2, and C3.

*The necrosis (necrotic zone)* was highest with diode laser with wavelength of 980 nm (group C3) (Figure 8), but there was no significant difference between C1, C2, and C3 (*p* = 0.223) (Table 2).

**Figure 9:**
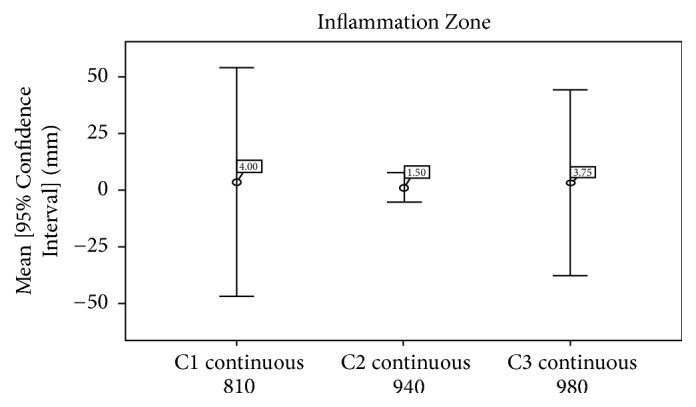
Lower inflammatory response in group C2 versus groups C1 and C3.

The comparison of* the inflammatory zones* between C1, C2, and C3 in this study was qualitative and appeared after 6 hours and was more marked at 15 hours than that at 6 hours with a high density of neutrophils. The inflammatory reaction was the lowest with C2 and equal with C1 and C3 (Figure 9). No statistical analysis of the inflammatory response was done due to the limited number of rabbits in each group (Table 2).

## 5. Discussion

Diode lasers have been used in dentistry since 1995. Today, several wavelengths of diode laser are used in dentistry: 655, 810, 940, 980, and 1.064 nm. The purpose of this study was to compare induced incisions on the ventral lingual mucosa of the rabbit using three diode lasers with wavelengths of 810, 940, and 980 nm in continuous mode.

The water absorption is highest with 980 nm lasers, followed by 940 nm and 810 nm. Also, hemoglobin absorption is highest with 980 nm laser and then 810 nm and is approximately the same as 940 nm. Melanin absorption is highest with 810 nm and then 940 nm and 980 nm lasers [4, 9–11, 15].

The diode lasers with 810, 940, and 980 nm wavelengths are ideally suited for soft tissue treatments because they are very well absorbed by hemoglobin and melanin, two great compounds in the oral soft tissues. Diode lasers are used to cut or destruct a tissue and to coagulate bleeding [4, 7, 10, 12, 15–17]. With these wavelengths, the penetration depth in biological tissues was relatively high at approximately 0.5–3 mm [6, 18].

Borchers [19] reported that cutting efficiency of a laser diode with a wavelength of 980 nm is greater than 810 nm. The 810 nm laser had a lower absorption by water, HbO_2_, and hemoglobin then 980 nm laser. The 980 nm laser can create more heat energy to the surface and deep penetration. The area of necrosis is larger and deeper. Bach et al. [20] reported in their in vitro study that there was no histological difference between the 810 nm diode laser and the 980 nm diode laser when both are used in the same mode. Using the laser diode in continuous mode, the cut was flattened and regular [19] and the cutting efficiency was not low.

In this study, the edges of incisions were well defined and were consistent with the 810 and 980 nm diode lasers used in a continuous mode but less with the 940 nm laser. The cutting efficiencies of the three lasers were identical. No statistically significant difference for the depth and the width of the incision was reported.

Other parameters other than wavelength can be involved in the cutting efficiency such as power, frequency, pulse duration, and fiber diameter. Parameters such as fluence and treatment time were related to tissue type (pigmented tissue), race, and blood circulation. The characteristics and parameters of the laser (output power, wavelength, terms transmission, type of optical fiber used, and the affinity with the target tissue) may condition the width and intensity of the thermal damage to tissue [5, 10, 19–22].

Our study aimed to compare the efficacy and tissue damage of different diode laser wavelengths on the oral tissue. The power, fiber diameter, target tissue, and operator were the same for the three lasers, which only varied by their wavelengths. The target was to realize an incision with 8 to 10 mm with the same number of passes and the same pressure.

In this study, the average depth was 4.70 +/− 3.67 for 810 nm laser, 4.69 +/− 3.95 mm for 980 nm laser, and 2.71 +/− 1.98 mm for 940 nm laser; but the difference was not statistically significant (*p* = 0.121), Whereas the width of the incision in this study was 2.97 +/− 1.36 mm for 810 nm laser, 2.94 +/− 1.56 mm for 940 nm laser, and 2.90 +/− 1.91 mm for 980 nm laser, with no statistical significance as well. Carbonization and necrotic and inflammatory zones were approximately the same for the three lasers, and the difference was not statistically significant.

Goharkhay et al. [15] reported that the horizontal and vertical tissue destruction dimension does not depend on the diameter of the fiber or the transmission mode (CW or pulsed) but only depends on the average power used.

Brochers [19] reported that the risk of carbonization of tissue destruction increases when the diameter of the fiber decreases. Carbonization, in turn, changes the absorption of the treated tissue and increases due to the dark color that absorbs the laser energy much better than light or bright colors. This means that there is more thermal energy applied to the surface and necrosis of adjacent structures is created. Thus, the parameters used may influence the degree of carbonization [10, 20, 23–26]. For Schäfer [27], the cause of the carbonization is therefore not recounted to the wavelength; rather it may be related to the characteristics of the laser timing. The laser used in continuous mode increases the tissue temperature more than pulsed mode. The burned tissue also exhibits a higher absorption coefficient than healthy tissue [8, 27]. The evaluation of the ability of healing after 14 days showed complete recovery with a power of 2 watts in continuous mode. However, Bryant et al. [28] found very slow healing with 3 watts of power. The incisions made with continuous mode showed an inflammatory response without delay in the healing process. Jin et al. [29] compared the healing of incisions made with a scalpel or a diode laser and an Er,Cr:YSGG laser on oral mucosa of guinea pigs and reported that there is less tissue damage with the use of a scalpel compared to an Er,Cr:YSGG laser and diode laser.

The diode laser is a valuable therapeutic tool for excisional and incisional biopsy in oral lesions but it induced significant thermal effects. However, from a clinical point of view, the limit of the specimen is essential, and the carbonization and the necrosis zone made the identification of the healthy tissue difficult. To submit a histological diagnosis, specimens must be intact and readable; the marginal integrity is very critical to evaluate the complete excision of malignant and premalignant tissue. There is considerable controversy about the use of diode laser in oral pathology. The specimens obtained with a diode laser have good histological readability [6, 15, 30]. However, characteristics and parameters of the laser (output power, wavelength, emission mode, type of optic fiber used, and affinity for the target tissue) can condition the width and the severity of thermal damage to tissue [31, 32].

## 6. Conclusion

Nowadays, no laser dentistry can completely replace conventional instruments while improving clinical outcomes. In oral pathology, where biopsy is the cornerstone of the diagnosis and therapy for many lesions, the laser can find its way through its various features (hemostatic, bactericidal, no pain, sutures, etc.). This study showed that the three diode lasers with different wavelengths (810 nm, 940 nm, and 980 nm) in continuous mode could be used for the incision of oral mucosa. In continuous mode, the edges of the incisions have been shown to be regular with the three types of lasers. The carbonization and necrotic and inflammatory zones were not statistically different between the three lasers.

The diode lasers are a favorable surgical option while executing incisions on the intraoral mucosa. The advantages of laser therapy include the minimal postoperative pain, the conservation of the specific site with minimal surgical invasion, and elimination of the need for a suture.

## Figures and Tables

**Table 1:** Results of the fourteen rabbits concerning the depth and width of the incision, the carbonization, and necrotic and inflammatory zones.

Rabbits	Sacrifice time	Groups	Depth	Width	Carbonization zone	Necrosis zone	Inflammatory zone	Edges
1	0 hours	C1						Regular
		C2	1.20	2.00	0.10	1.00		Regular
		C3	3.80	3.00	0.10	1.50		Regular

2	0 hours	C1	6.50	0.60	0.05	0.30		Regular
		C2	3.50	2.00	0.10	0.50		Irregular
		C3	8.00	7.00	0.20	1.00		Regular

3	0 hours	C1	14.00	3.50	0.10	0.50		Regular
		C2	2.50	3.50	0.10	0.50		Regular
		C3	14.00	2.50	0.10	0.50		Regular

4	0 hours	C1	3.00	3.00	0.10	0.70		Regular
		C2	2.50	5.50	0.10	1.00		Regular
		C3	5.00	3.00	0.10	0.80		Regular

5	0 hours	C1	5.00	3.00	0.10	0.40		Regular
		C2	6.00	2.50	0.10	0.50		Irregular
		C3	9.50	2.00	0.10	0.50		Regular

6	1 hour	C1	1.40	2.00	0.10	0.50		Regular
		C2	1.20	1.50	0.10	0.30		Regular
		C3	0.00	0.00	0.00	0.00		

7	2 hours	C1	2.50	2.00	0.10	0.70		Regular
		C2	0.00	0.00	0.00	0.00		Regular
		C3	3.00	1.50	0.10	1.00		Regular

8	2 hours	C1	2.00	2.50	0.10	0.30		Regular
		C2	6.00	3.00	0.20	1.00		Irregular
		C3	2.50	3.00	0.10	0.40		Regular

9	2 hours	C1	9.00	6.00	0.20	0.50		Regular
		C2	4.80	3.00	0.50	0.50		Regular
		C3	2.50	3.50	0.20	0.50		Regular

10	2 hours	C1	4.00	4.00	0.10	0.50		Regular
		C2	1.20	3.00	0.10	0.50		Regular
		C3	3.80	2.00	0.20	0.50		Regular

11	2 hours	C1	3.00	4.00	0.10	1.00		Regular
		C2	1.00	5.00	0.10	1.00		Regular
		C3	4.00	2.50	0.10	1.00		Regular

12	6 hours	C1	4.50	3.00	0.10	0.30	0.00	
		C2	1.50	1.80	0.10	0.30	1.00	
		C3	1.50	1.80	0.10	0.30	0.50	

13	15 hours	C1	1.50	2.00	0.00	2.00	8.00	
		C2	2.30	4.50	0.00	2.00	2.00	
		C3	2.50	6.00	0.00	3.00	7.00	

14	14 days	C1	0.00	0.00	0.00	0.00	0.00	
		C2	0.00	0.00	0.00	0.00	0.00	
		C3	0.00	0.00	0.00	0.00	0.00	

**Table 2:** Comparison of the three groups for the depth, width, carbonization, and necrotic zone.

	C1	C2	C3	*p* value^*∗*^
Depth	4.70 +/− 3.67	2.71 +/− 1.98	4.69 +/− 3.95	0.121
Width	2.97 +/− 1.36	2.94 +/− 1.56	2.90 +/− 1.91	0.376
Carbonization	0.10 +/− 0.05	0.13 +/− 0.13	0.11 +/− 0.07	0.692
Necrotic zone	0.64 +/− 0.47	0.68 +/− 0.52	0.79 +/− 0.76	0.223
Inflammation	4.00 +/− 4.00	1.50 +/− 1.00	3.75 +/− 3.25	-

^*∗*^Friedman test.

## References

[1] Maiman T. H. (1960). Stimulated optical radiation in Ruby.

[2] Pirnat S. (2007). Versatility of an 810 nm diode laser in dentistry: an overview.

[3] Goldstep F. (2009). Soft tissue diode laser: Where have you been all my life?.

[4] Goldstep F., Freedman G. (2012). Diode laser: The soft-tissue handpiece.

[5] Romanos G., Nentwig G.-H. (1999). Diode laser (980 nm) in oral and maxillofacial surgical procedures: Clinical observations based on clinical applications.

[6] Aoki A., Mizutani K., Takasaki A. A. (2008). Current status of clinical laser applications in periodontal therapy.

[7] Baxter W. T., Mironov S. F., Zaitsev A. V., Jalife J., Pertsov A. M. (2001). Visualizing excitation waves inside cardiac muscle using transillumination.

[8] White J. M., Chaudhry S. I., Kudler J. J., Sekandari N., Schoelch M. L., Silverman S. (1998). Nd: YAG and CO2 laser therapy of oral mucosal lesions.

[9] Moritz A., Gutknecht N., Doertbudak O. (1997). Bacterial reduction in periodontal pockets through irradiation with a diode laser: A pilot study.

[10] Moritz A., Schoop U., Goharkhay K. (1998). Treatment of periodontal pockets with a diode laser.

[11] Coluzzi D. J. (2010). An overview of laser in dentistry, fundamentals of lasers.

[12] Coluzzi D. J. (2010). Absorption of laser by dental tissue.

[13] Manni J. G. (2004).

[14] Alamarguy C. (2011).

[15] Goharkhay K., Moritz A., Wilder-Smith P. (1999). Effects on oral soft tissue produced by a diode laser in vitro.

[16] Walinski C. J. (2004). Irritation fibroma removal: A comparison of two laser wavelengths.

[17] Adams T. C., Pang P. K. (2004). Lasers in aesthetic dentistry.

[18] Moritz A., Beer F., Goharkhay K. (2006).

[19] Borchers R. (2008).

[20] Bach G., Koch H. K., Hellerich U., Venzke T. (2008). Konventionelle Diodenlaser versus Hochpulstechnik.

[21] Horch H. H. (2002).

[22] Hartmann H. J., Bach G. (1997). Diodenlaser- Oberflächen- Dekontamination in der Periimplantitis therapie.

[23] Black P. (2007). Der Dentallaser in der oralen Chirurgie-Masterthese-Teil2.

[24] Saleh H. M., Saafan A. M. (2007). Excision biopsy of tongue lesions by diode laser.

[25] Janda P., Sroka R., Mundweil B., Betz C. S., Baumgartner R., Leunig A. (2003). Comparison of thermal tissue effects induced by contact application of fiber guided laser systems.

[26] Welch A. J. (1984). The Thermal Response of Laser Irradiated Tissue.

[27] Schäfer O. (2011). The evolution of the diode laser.

[28] Bryant G. L., Davidson J. M., Ossoff R. H., Garrett C. G., Reinisch L. (1998). Histologic study of oral mucosa wound healing: a comparison of a 6.0- to 6.8-*µ*m pulsed laser and a carbon dioxide laser.

[29] Jin J.-Y., Lee S.-H., Yoon H.-J. (2010). A comparative study of wound healing following incision with a scalpel, diode laser or Er,Cr:YSGG laser in guinea pig oral mucosa: A histological and immunohistochemical analysis.

[30] Capodiferro S., Maiorano E., Loiudice A. M., Scarpelli F., Favia G. (2008). Oral laser surgical pathology: a preliminary study on the clinical advantages of diode laser and on the histopathological features of specimens evaluated by conventional and confocal laser scanning microscopy..

[31] Eversole L. R. (1997). Laser artifacts and diagnostic biopsy.

[32] White J. M., Goodis H. E., Rose C. L. (1991). Use of the pulsed Nd:YAG laser for intraoral soft tissue surgery.

